# The clinical significance of aldosterone synthase deficiency: report of a novel mutation in the *CYP11B2* gene

**DOI:** 10.1186/1472-6823-14-29

**Published:** 2014-04-03

**Authors:** Elaine Hui, Matthew CW Yeung, Pik To Cheung, Elaine Kwan, Louis Low, Kathryn CB Tan, Karen SL Lam, Angel OK Chan

**Affiliations:** 1 Department of Medicine, The University of Hong Kong, Queen Mary Hospital, Pokfulam Road, Pokfulam, Hong Kong; 2 Department of Pathology, The University of Hong Kong, Queen Mary Hospital, Pokfulam Road, Pokfulam, Hong Kong; 3 Department of Pediatrics and Adolescent Medicine, The University of Hong Kong, Queen Mary Hospital, Pokfulam Road, Pokfulam, Hong Kong

**Keywords:** Aldosterone synthase deficiency, Salt-wasting, Hypoaldosteronism

## Abstract

**Background:**

Aldosterone synthase (*CYP11B2*) deficiency is a rare autosomal recessive disorder, usually presenting with severe salt-wasting in infancy or stress-induced hyperkalaemia and postural hypotension in adulthood. Neonatal screening for congenital adrenal hyperplasia, another cause of salt wasting, using 17-hydroxyprogesterone measurement would fail to detect aldosterone synthase deficiency, a diagnosis which may be missed until the patient presents with salt-wasting crisis. Due to this potential life-threatening risk, comprehensive hormonal investigation followed by genetic confirmation for suspected patients would facilitate clinical management of the patient and assessment of the genetic implication in their offspring.

**Case presentation:**

We describe a 33-year old Chinese man who presented in infancy with life-threatening hyponatraemia and failure to thrive, but remained asymptomatic on fludrocortisone since. Chromosomal analysis confirmed a normal male karyotype of 46, XY. Plasma steroid profile showed high plasma renin activity, low aldosterone level, and elevated 18-hydroxycorticosterone, compatible with type 2 aldosterone synthase deficiency. The patient was heterozygous for a novel *CYP11B2* mutation: c.977C > A (p.Thr326Lys) in exon 3. He also carried a heterozygous mutation c.523_525delAAG (p.Lys175del) in exon 6, a known pathogenic mutation causing aldosterone synthase deficiency. Sequencing of *CYP11B2* in his parents demonstrated that the mother was heterozygous for c.977C > A, and the father was heterozygous for c.523_525delAAG.

**Conclusion:**

Although a rare cause of hyperreninaemic hypoaldosteronism, aldosterone synthase deficiency should be suspected and the diagnosis sought in patients who present with life-threatening salt-wasting in infancy, as it has a good long-term prognosis when adequate fludrocortisone replacement is instituted. To our knowledge, this is the first Chinese patient in which the molecular basis of aldosterone synthase deficiency has been identified.

## Background

Aldosterone is the main mineralocorticoid hormone in humans that regulates sodium excretion and intravascular volume. It acts via distal renal tubules and cortical collecting ducts by increasing sodium reabsorption from and potassium excretion into the urine. It is synthesized by aldosterone synthase (*CYP11B2*), an enzyme encoded by *CYP11B2*, expression of which is almost entirely confined to the adrenal cortex and exclusively in the zona glomerulosa layer. Aldosterone synthase is a cytochrome P450 enzyme that catalyses the final 3 steps of aldosterone biosynthesis: first by the hydroxylation of deoxycorticosterone (DOC) at position 11β to form corticosterone (B), then the hydroxylation at position 18 to form 18-hydroxycorticosterone (18OHB), and lastly the oxidation at position 18 to aldosterone [1]. Isolated deficiencies of aldosterone biosynthesis are caused by inactivating mutations in the *CYP11B2* gene [2,3]. Aldosterone deficiency leads to excessive sodium excretion and potassium retention, resulting in hyponatraemia, hyperkalaemia, and metabolic acidosis. Cases of aldosterone synthase deficiency (ASD) have been identified in Iranian Jews, Europeans and North Americans [4]. In Asians, it has been reported in Thai, Japanese and Indian individuals [5,6]. To our knowledge, there has been no report of Chinese cases with confirmed genetic analysis. Here, we describe a case of aldosterone synthase deficiency in a Chinese man and results of *CYP11B2* analysis in the patient and his family.

## Case presentation

### Case report

The index patient, at the time of this report aged 33 years old, was delivered normally at term with a birth weight of 3.1 kg. He had repeated vomiting with poor feeding and failure to thrive at 5 weeks of age. He was found to have hyponatraemia and was started on cortisone acetate 2.5 mg three times daily and fludrocortisone 50 μg daily on the presumptive diagnosis of congenital adrenal hyperplasia. He had normal male phenotype and chromosomal analysis confirmed a normal male karyotype of 46, XY. He was evaluated with supervised treatment withdrawal by 6 months old. 24-hour urinary ketosteroid was normal but plasma renin activity was high and aldosterone was low, suggestive of hypoaldosteronism. Cortisone acetate was subsequently stopped. Plasma steroid profiling, which was performed after withholding therapy for a week at the age of 14.5 years old, gave characteristic findings of type 2 ASD (Table 1). Developmental milestones, electrolytes and blood pressure were normal while on fludrocortisone 100 μg daily. Both his unrelated parents and his older brother were phenotypically normal.

**Table 1:** Plasma steroid profile of the patient

	**Basal (nmol/L)**	**Post-ACTH stimulation (nmol/L)**		
**Aldosterone**	0.166 (0.39-2.44)	0.19 (0.01 – 0.55)		
**18-OH-Corticosterone (18-OHB)**	7.06 (0.56 – 1.46)	12.1 (0.83 – 5.5)		
**Corticosterone**	44.4 (0.14 – 14.23)	151.8 (34.0 - 208.4)		
**18-OH-deoxycorticosterone**	1.64 (0.13-0.72)	5.28 (0.58 – 6.1)		
**Deoxycorticosterone**	0.57 (0.09 – 0.57)	2.36 (0.27 – 1.35)		
**Progesterone**	1.94 (0.095 – 3.15)	2.96 (0.52 – 2.55)		
**17-hydroxyprogesterone**	3.09 (0.24 – 4.93)	15.2 (1.88 – 10.96)		
**11-Deoxycortisol**	2.22 (0.15-2.58)	14.5 (1.36 - 9.44)		
**Cortisol**	274.3 (59.9 - 480)	721.5 (317.3 – 979.3)		
**Cortisone**	161.2 (6.37-123.6)	59.9 (34.8 – 79.7)		
**Ratio**				
**B/18-OHB**	6	12		
**18-OHB/ aldo**	43	63		

Plasma steroid profile showed low aldosterone level, elevated 18-hydroxycorticosterone (18-OHB), low corticosterone/18-OHB and high 18-OHB/aldosterone ratios, compatible with type 2 aldosterone synthase deficiency.

### Genetic analysis

**Figure 1:**
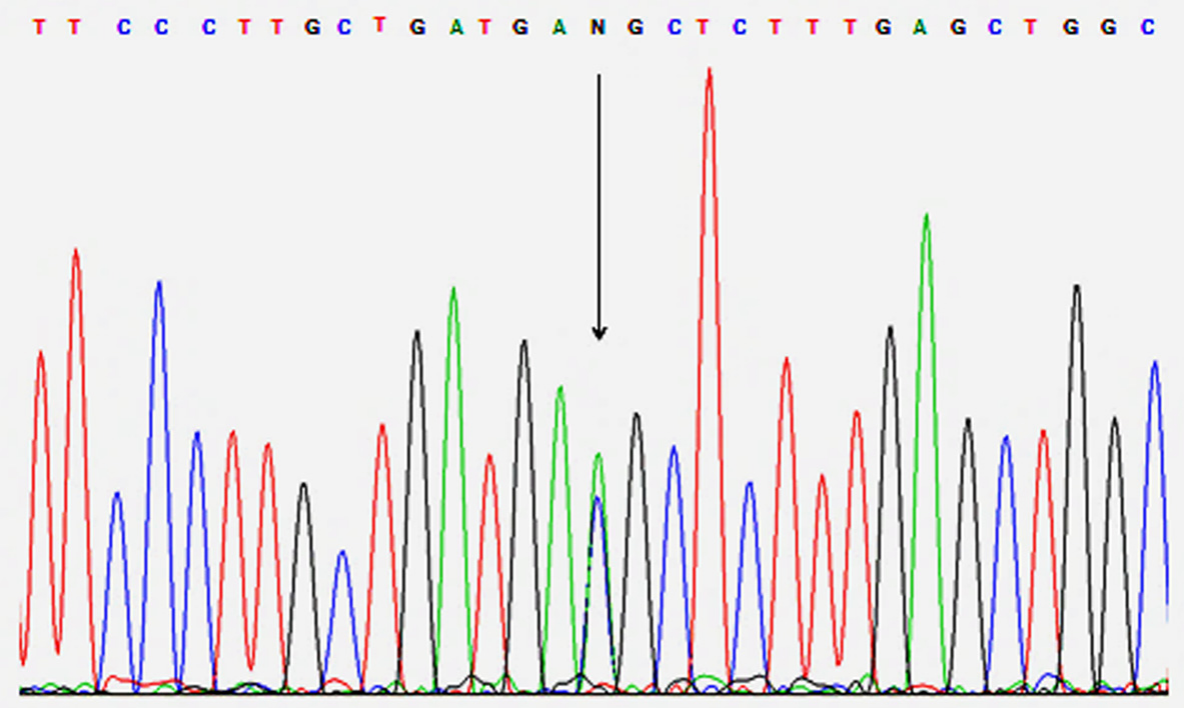
**Electropherogram of segment of *CYP11B2 *showing the heterozygous c.977C > A mutation.** The mutation site is denoted by the letter N and is indicated by an arrow.

During family planning, genetic analysis was performed to ascertain the diagnosis at the age of 33 years. Blood samples were obtained and genomic DNA extracted from the patient, his brother and both non-consanguineous parents with informed consent. The nine coding exons and flanking intronic regions (15 bp) of the *CYP11B2* gene were amplified by polymerase chain reaction on the patient’s genomic DNA, followed by direct DNA sequencing using primers and conditions as previously described [5]. Targeted mutational analysis was also performed on both parents and his brother’s genomic DNA. The patient and family members’ DNA sequences were compared to NCBI Reference Sequences, NG_008374.1, NM_000498.3 and NP_000489.3. The affected patient carried a novel heterozygous missense mutation: c.977C > A (p.Thr326Lys) in exon 3 (Figure 1), which has not previously been reported. He also carried a heterozygous mutation c.523_525delAAG (p.Lys175del) in exon 6, a known pathogenic frameshift mutation causing ASD [7]. Sequencing of *CYP11B2* in the parents demonstrated that the mother was heterozygous for c.977C > A (p.Thr326Lys) and the father was heterozygous for c.523_525delAAG (p.Lys175del). Neither mutation was detected in the patient’s brother.

## Discussion

We describe a Chinese man who presented with severe salt-wasting in infancy and hyperreninaemic hypoaldosteronism, and in whom genetic analysis confirmed aldosterone synthase deficiency. He remained clinically well in adulthood with normal electrolytes and blood pressure whilst on fludrocortisone.

Inadequate aldosterone production results in decreased renal sodium reabsorption and potassium excretion in ASD. Clinical severity varies, with the most severe forms presenting in infancy. Affected infants usually develop vomiting, dehydration, hypovolaemia, and failure to thrive at a few days to weeks following delivery [8]. However, salt-losing crisis in isolated ASD is usually indistinguishable clinically from other forms of defective steroid biosynthesis, such as 21-hydroxylase deficiency in congenital adrenal hyperplasia. Therefore, life-threatening salt-wasting in affected infants is usually treated initially with both hydrocortisone and fludrocortisone pending steroid hormone analysis [9]. Phenotypically, infants with ASD have normal external genitalia [5]. Hydrocortisone can be withdrawn once characteristic steroid profile or genetic mutations of *CYP11B2* gene are confirmed. A typical biochemical profile of this disorder includes hyponatraemia, hyperkalaemia, raised plasma renin activity, undetectable or low aldosterone level, and normal or elevated cortisol levels.

Aldosterone synthase deficiency has been classified based on biochemical phenotypes: in type 1 ASD, the enzymatic *CYP11B2* activity is completely abolished. Affected individuals have low to normal levels of 18OHB, and undetectable to low levels of plasma aldosterone or its urinary metabolite, tetrahydroaldosterone [10]. In type 2 deficiency, the mutations in the *CYP11B2* gene only decrease 18-hydroxylase and 18-oxidase activities, but not 11β-hydroxylase activity. It differs from type 1 in that 18-OHB levels are markedly elevated, whilst plasma aldosterone levels and urinary tetrahydroaldosterone are low [7]. However, it is now recognised that these biochemical phenotypes have clinical, hormonal and genotypic overlapping features [11] and that type 1 and type 2 ASD would be better considered a continuous spectrum of the same disease [5].

As clinical severity gradually improves with age, affected adults are usually asymptomatic despite no mineralocorticoid therapy [5,12]. However, termination of mineralocorticoid may lead to postural hypotension and hyperkalaemia when triggered by stress due to dehydration or reduced salt intake in some affected adults [12]. Generalised weakness with marked hyperkalaemia and dehydration has been reported as the first presentation in a middle-aged man after concurrent institution of indapamide for hypertension and bowel preparation for barium enema. Interestingly, further questioning revealed a past history of vomiting and failure to thrive in early infancy that resolved without any mineralocorticoid replacement [13]. Therefore, detailed childhood history would be a helpful clue in suspected ASD case presenting in adulthood. Possible mechanisms for reduced clinical severity of ASD with advancing age include increasing sensitivity to mineralocorticoid action and sodium intake with age. It has been shown that mineralocorticoid receptor expression in human kidneys begins with low levels in late gestation and rises progressively after birth [14]. Another potential mechanism is age-dependent impaired 11β-hydroxysteroid dehydrogenase type 2 activity, leading to greater cortisol availability for the mineralocorticoid receptor with age [15].

Neonatal screening for CAH using 17-hydroxyprogesterone measurement would fail to detect ASD, as infants with ASD usually have normal [12] or slightly elevated basal and stimulated 17-hydroxyprogesterone [9]. The diagnosis of ASD may therefore be missed until the patient presents with salt-wasting crisis. Therefore, comprehensive hormonal investigation followed by genetic confirmation for suspected adults and their respective partners would be useful to assess the genetic implication in their offspring and to minimize the potential life-threatening risk.

**Figure 2:**
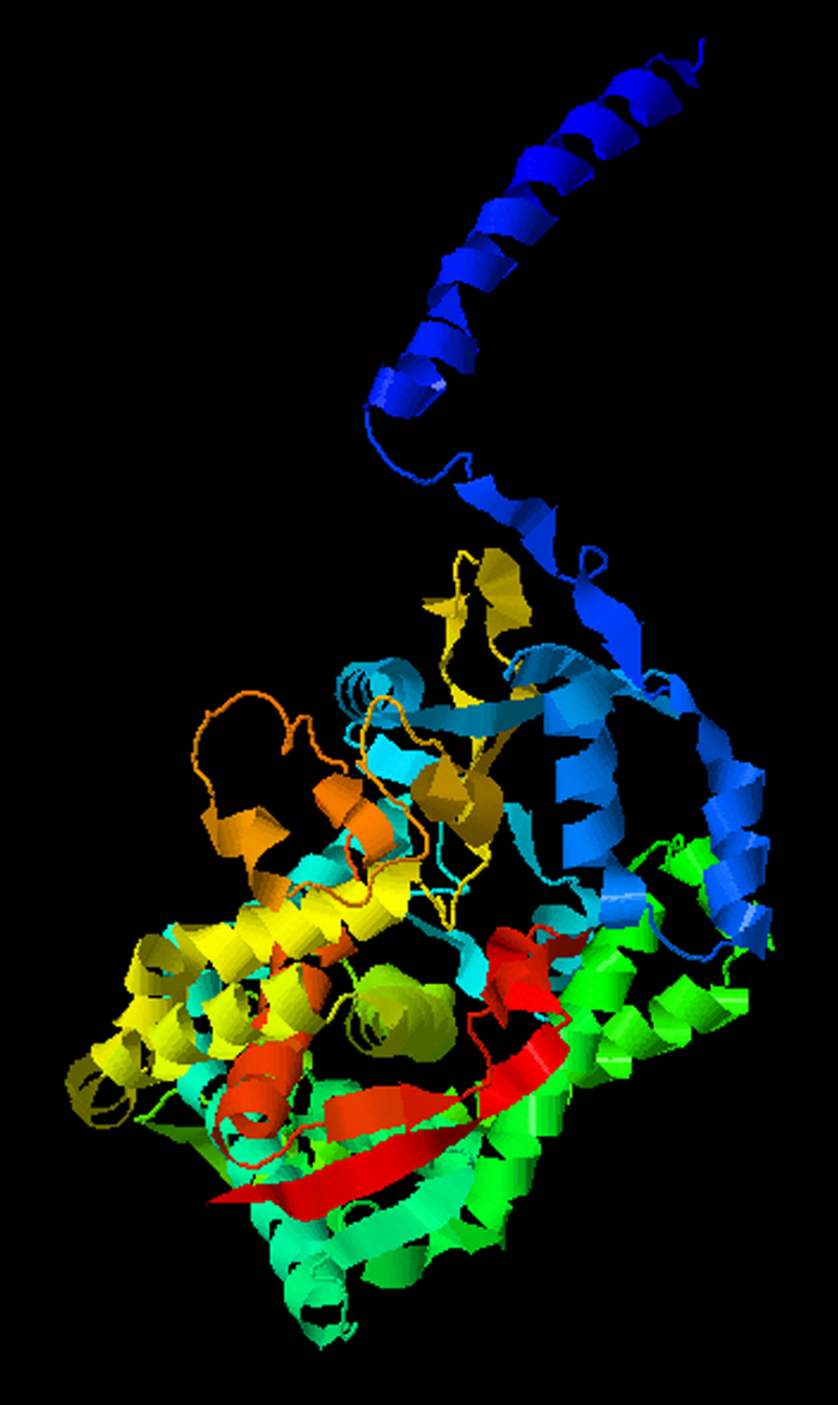
**3-D model of the *CYP11B2 *mutant with the novel missense c.977C > A mutation.** The threonine residue in codon 326 is located in the I-helix of *CYP11B2* protein, which is implicated in 18-hydroxylation/oxidation due to the clusters of *CYP11B2* isoform-specific residues in that region. In particular, the alanine residue in position 320 is required for methyloxidase reaction and the p.Thr318Met in the I-helix is a known deleterious mutation causing aldosterone synthase deficiency. This structural information and functional correlation suggest that the I-helix region, where the novel variant c.977C > A (p.Thr326Lys) is located, is important for the enzyme activity of *CYP11B2*.

Compound heterozygosity has been reported when phenotypically unaffected parents contribute to each allele with a heterozygous mutation, rendering phenotypes of ASD type 1 in the affected offspring [6,10]. Our current case inherited one of the heterozygous mutations c.977C > A (p.Thr326Lys) maternally, and the other c.523_525delAAG (p.Lys175del) paternally. The unaffected parents are heterozygous for their respective mutations, in accordance with an autosomal recessive inheritance. *In silico* protein function prediction using PolyPhen-2 and Align GVGD both predict the novel missense variant c.977C > A (p.Thr326Lys) to be damaging. Multiple sequence alignment of the *CYP11B2* gene also showed that the threonine residue is highly conserved across multiple mammal species (Table 2). The threonine residue in codon 326 is located in the I-helix of *CYP11B2* protein (Figure 2). A previous study mapping the amino acid difference between *CYP11B2* and *CYP11B1* showed that *CYP11B2* isoform-specific residues are clustered around the H-helix and in the I-helix and are essential for 18-hydroxylation/oxidation [16]. In particular, the alanine residue in position 320 in *CYP11B2* is required for methyloxidase reaction. Also, p.Thr318Met in the I-helix is a known deleterious mutation causing aldosterone synthase deficiency [11]. This structural information and functional correlation suggest that the I-helix region, where the novel variant c.977C > A (p.Thr326Lys) is located, is important for enzyme activity of CYP11B2. Taking all the aforementioned factors into consideration, the variant c.977C > A (p.Thr326Lys) is likely to be pathogenic, in keeping with our case.

**Table 2:** **Multiple sequence alignment of a segment of the *CYP11B2 *** **gene**

**Homo sapiens**	GSVDTTAFPLLM**T**LFELARNPDV
**Canis lupus**	GSVDTTAYPLWM**T**LFELARNPDV
**Mus musculus**	GSVDTTAIPLVM**T**LFELARNPDV
**Macaca mulatta**	GSVDTTAFPLLM**T**LFELARNPDV
**Rattus norvegicus**	GSVDTTAIPLVM**T**LFELARNPDV

Codon 326 of the *CYP11B2* gene in *homo sapiens* and the corresponding codon in other species are in bold phase. The threonine residue at codon 326 (in bold) is highly conserved across different mammal species.

Homozygous p.Lys175del (previously described as homozygous deletion of codon 173 before the use of the latest HGVS nomenclature) has been reported in a girl presenting with salt-wasting during infancy [7]. *CYP11B2* is polymorphic at this position, encoding arginine or lysine. Amino acid residue 173 is positioned in α-helix D, and the secondary structure of aldosterone synthase was presumed to be altered by the single amino acid deletion. Indeed, using fission yeast system, Tin *et al.* demonstrated that the deletion mutation at codon 173 displayed markedly reduced 11-β-hydroxylation activity (16.3%), and no 18-hydroxylation and 18-oxidation activities [17].

## Conclusion

Aldosterone synthase deficiency is a rare cause of hyperreninaemic hypoaldosteronism and its genetic and molecular basis is more heterogeneous than previously described. It should be suspected in infants without virilisation presenting with salt-wasting or in adults presenting with stress-induced hyperkalaemia and a history of failure to thrive in childhood. Our case illustrates the clinical significance to recognize this condition as it has a good long-term prognosis when adequate fludrocortisone replacement is instituted.

## Consent

Written informed consent was obtained by the patient for the publication of this case report and any accompanying images. A copy of the written consent is available for review by the Editor of this journal.

## Competing interests

The authors declare that they have no competing interests.

## Authors’ contributions

EH and MY drafted the manuscript. PTC, EK, LL, MY and AC performed the biochemical and genetic analysis. KT, KL and AC revised the manuscript critically. All authors read and approved the final manuscript.

## Pre-publication history

The pre-publication history for this paper can be accessed here:

http://www.biomedcentral.com/1472-6823/14/29/prepub
